# Association between Cognition and Serum Insulin-Like Growth Factor-1 in Middle-Aged & Older Men: An 8 Year Follow-Up Study

**DOI:** 10.1371/journal.pone.0154450

**Published:** 2016-04-26

**Authors:** Shankar Tumati, Huibert Burger, Sander Martens, Yvonne T. van der Schouw, André Aleman

**Affiliations:** 1 Department of Neuroscience, University Medical Center Groningen, University of Groningen, The Netherlands; 2 Department of General Practice, University Medical Center Groningen, Groningen, The Netherlands; 3 Interdisciplinary Center for Psychopathology and Emotion Regulation, University Medical Center Groningen, Groningen, The Netherlands; 4 Julius Center for Health Sciences and Primary Care, University Medical Center Utrecht, Utrecht, The Netherlands; Albert Einstein College of Medicne, UNITED STATES

## Abstract

Low levels of insulin-like growth factor-1 (IGF-1), an essential neurotrophic factor, have been associated with worse cognitive function in older adults. However, few studies have assessed the prospective association of serum IGF-1 with cognitive function. We aimed to determine the association between serum IGF-1 on concurrent and prospective cognitive function in a population sample of men aged 40–80 years. Blood samples were assessed for IGF-1 levels at baseline and neuropsychological assessments were performed at baseline (n = 400) and at follow-up after a mean duration of 8.3 years (n = 286). Linear regression analyses were carried out to determine the associations between quintiles of IGF-1 and cognitive function at the baseline and follow-up visits. Results showed that those in the top quintile of IGF-1 had lower processing capacity and global cognition scores at follow-up after controlling for cognitive function at baseline and other confounding factors. Additional analyses exploring associations with IGF-1 separately in middle-aged and older participants, and with quartiles of IGF-1 produced similar results. In those older than 60 years, high IGF-1 levels were also associated with lower baseline processing capacity. These results suggest that high IGF-1 levels are associated with worse long-term cognition in men. Together with past studies, we suggest that both, high and low levels of IGF-1 may be associated with poor cognitive function and that optimum levels of IGF-1 (quintile 2 and 3 in current study) may be associated with better cognitive function.

## Introduction

Cognitive decline is common with increasing age, and leads to a reduced quality of life and capacity for independent daily functioning [1]. Though characteristic of old age, subtle worsening of cognitive performance begins much earlier even in healthy individuals [2], suggesting that biological changes underlying cognitive decline begin at a younger age. Identifying such early biological changes will improve understanding of the mechanisms that underlie cognitive decline and assist in development of interventions that enhance quality of life in old age.

Insulin-like growth factor-1 (IGF-1) is an essential neurotrophic factor that is produced both peripherally and in the brain. Peripheral levels of IGF-1, measured in the serum, have been previously associated with cognitive function [3,4]. Studies in older individuals suggest that low levels of IGF-1 are associated with reduced processing capacity [5], fluid intelligence [6], and verbal expression [7]. Prospective studies have suggested that lower levels of IGF-1, especially below threshold levels are associated with poor cognition [8–10] (for review see [11]). In short, adequate levels of serum IGF-1 may be necessary for normal cognitive functioning.

As cognitive decline begins early, it is important to study cognitive changes occurring at a younger age. However, previous studies on IGF-1 have mostly assessed cognitive functioning in older individuals. In a small number of studies where this was not the case, cognition was measured using brief neuropsychological assessments. In addition, most studies were limited by short follow-up durations. As worsening of cognitive performance is gradual, especially in relatively younger individuals, cognitive assessments over longer time periods are necessary to enhance sensitivity for detecting such gradual changes.

This study aimed to investigate the association between serum IGF-1 concentrations and cognitive functioning. Keeping in mind the above-mentioned limitations, this study was conducted using a large prospective population cohort of middle-aged and older men. Detailed cognitive assessments were conducted at two time points approximately eight years apart.

## Methods

The parent study, conducted in Utrecht, the Netherlands, is a prospective multi-disciplinary population-based longitudinal single-center study of independently living middle-aged and older men in the age range of 40–80 years [12]. In 2001–2002, participants were recruited by requesting female participants of previous studies conducted at the center to suggest male volunteers between 40 and 80 years old. Invitation letters were sent to 770 potential participants, of which 240 volunteered to participate in the study. In addition, 1230 males were randomly selected from community registers and sent invitations, of which 390 men volunteered for the study. Volunteers who were unable to live independently or were unable to visit the health center were excluded (n = 16). The remaining group of 614 volunteers were divided into four 10-year age groups and random samples of 100 participants were drawn from each age group to create a stratified sample of 400 men.

Between 2010 and 2011, all participants still living (N = 351) were invited for re-examination to the study center. A total of 286 men (participation rate = 71.5%) were re-examined. Reasons for non-participation were emigration (n = 4), inability to visit the study center (n = 31), not interested or non-response (n = 30). The mean duration between the two assessments was 8.3 (standard deviation: 0.4) years. The mean age (range) of the participants at baseline was 60.2 (40–80) years and mean age at follow-up was 67.2 (48–88) years. The study was approved by the Institutional Review Board of Utrecht University and conformed to the principles of the Declaration of Helsinki. Written informed consent was obtained from all participants for both assessments.

### Cognitive Assessment

Trained research personnel conducted the following seven tests. The Mini Mental State Examination (MMSE), a measure of global cognition, consists of 20 questions with a maximum score of 30 [13]. The Rey Auditory Verbal Learning Test (RAVLT) assesses verbal episodic memory by measuring the rate and capacity for learning a list of words. For this, the examiner recited a 15-word list at the rate of one word per second. The participant was asked to recall as many words as he could remember. This sequence was repeated five times to obtain a total score representing immediate recall. After 30 minutes the participant was once again asked to recall the words in the list. The number of correctly remembered words represented the delayed recall score. Participants were then asked to recognize the 15 test words from a list of 30 words. The number of correct answers comprised the recognition score with a maximum score of 30 [14]. The Doors Test was used to assess visual recognition. Participants were shown pictures of doors (total of 24) and were asked to recognize the correct (previously shown) ones amongst a picture panel with four doors each [15]. The Digit Symbol Substitution Test (DSS) is a sensitive test for cognition that primarily assess information processing capacity. Participants were presented with a reference key comprising of symbols paired with numbers and asked to pair as many symbols with the corresponding number in 90 seconds. The reference key of digit-symbol pairing was visible during the test [16]. The Digit Span Test measures working memory and information processing ability. Scores consisted of the longest sequence of numbers (maximum 8) that a participant could recall in forward (digit span forward) and reverse (digit span backward) order [16]. For the Trail Making Test (TMT), participants were asked to connect numbers (TMT A1) and letters (TMT A2) sequentially. For TMT part B (TMT B), numbers and letters were both presented and the participants were asked to alternate between connecting numbers and letters, thus forming two sequences. TMT A1 & A2 measure processing capacity whereas TMT B measures executive function. The Verbal Fluency Test (VF), measuring cognitive control, consists of (a) naming as many nouns as possible starting with ‘n’ and ‘a’ and, (b) naming as many animals and occupations as possible in sixty seconds.

Composite scores were computed by summing the standardized scores of individual tests into three cognitive domains: memory performance (combining scores from the Doors Test, RAVLT scores of Immediate recall, Delayed recall and Recognition), processing capacity and speed (combining scores from the Digit Span—forward and—backward, DSS, and TMT-A1 & A2), and executive function (combing scores from VF and TMT part B) [17]. A higher score on the tests denotes better performance except in case of the TMT, for which the sign of the Z score was reversed to denote better performance.

### IGF-1 measurement

At baseline, blood samples were collected between 8 and 10 am after an overnight fast. Platelet-free serum was obtained by centrifugation and stored at -80°C. IGF-1 was measured using an immunometric technique on an Advantage Chemiluminescense System (Nichols Institute Diagnostics, San Juan Capistrano, USA). The lower limit of detection was 6.0 ng/mL and inter-assay variation was 9.7, 6.4 and 5.1% at 51, 183 and 424 ng/L, respectively (n = 280).

### Covariates

During the initial visit, factors likely to affect cognitive function or IGF-1 were recorded among other details [12], including age, level of education and smoking history. Education (highest level achieved), was assessed with the 7-point Verhage scale where 1 = less than 6 years of elementary school, 2 = elementary school, 3 = elementary school and less than 3 years of secondary education, 4 = elementary school and 3 years of secondary education, 5 = elementary school and 4 years of lower general secondary education, 6 = pre-university education and higher vocational education, 7 = university and technical college [18]. Height and weight were recorded and body mass index values (BMI) were calculated (dividing weight (kg) by the square of height (in meters)). Physical activity was assessed using the Physical activity questionnaire [19]. Fasting blood glucose levels were assessed with a GlucoTouch reflectometer (LifeScan Benelux, the Netherlands), which uses a reagent strip glucose oxidase method.

### Statistical analysis

Previous studies have indicated that the association between serum IGF-1 and cognition may be present in a threshold dependent manner [8]. Therefore, we divided IGF-1 levels into quintiles. MMSE scores measured at baseline and follow-up were log transformed to achieve a normal distribution. Composite scores of memory performance, processing capacity, executive function, and log MMSE scores were used as outcome variables. Demographic variables characterizing the study sample were calculated according to quintiles of IGF-1, and compared using analysis of variance and chi-squared tests.

At baseline, composite cognitive scores for 3 cases and glucose measurements for 4 cases were missing. At follow-up, the rate of missingness across the quintiles of IGF-1 was: Q1–28.2%, Q2–25.3%, Q3–28.8%, Q4–33.8%, and Q5–26.3%. Of the 286 men at follow-up, complete data for composite cognitive scores ranged from 66% to 71.5%.

To avoid loss of statistical power due to exclusion of data of those lost to follow-up, multiple imputation by chained equations was performed. We assumed that data were missing at random (MAR) or missing completely at random (MCAR). Logistic regression was performed to determine if missingness for the main variables of interest could be predicted by baseline measurements of variables that could potentially lead to loss of follow-up. These included age, education, occupational level, composite cognitive scores, MMSE scores, Dutch version of the Adult reading test (a measure of pre-morbid intelligence), presence of a chronic disease, number of chronic diseases, smoking history (in pack-years), alcohol consumption (in units/week), physical activity score, marital status, whether living alone or not, and measures of physical health such as heart rate, systolic and diastolic blood pressure, BMI, serum levels of IGF-1, glucose, cholesterol, lipids, homocysteine, cortisol, and testosterone. Among these variables, significant predictors of missingness included age, presence and number of chronic diseases, occupational level, marital status, homocysteine levels, heart rate, and executive function score at baseline. These variables together explained 32% to 39% of the variance (Nagelkerke R^2^) for each cognitive score, partially substantiating the assumption of MAR. Thus, use of an imputed dataset for analyses would improve the validity of the results, as compared to a complete case analysis. All variables in the main model of analyses and significant predictors of missingness were included in the imputation model. In place of quintiles of IGF-1 used in the main model, serum IGF-1 values were included in the MI models. MI were performed using fully conditional specification wherein separate models specify the distribution for each variable with missing data [20]. Predictive mean matching was used to impute missing data as the variables were continuous in nature. To achieve adequate results, the number of imputations is recommended to be equal to the percentage missingness [21]. Accordingly, 30 imputed datasets (with 10 iterations each) were constructed for the rate of 28.5% missingness. Results from each imputed dataset were pooled according to Rubin’s method for multiple imputation inference [22].

Linear regression was performed using the imputed dataset to determine the association of quintiles of IGF-1 with cognitive performance at baseline and follow-up. In statistical models, quintile 5 was used as the reference group and covariates included age, level of education, smoking, BMI, blood glucose levels, and physical activity. In models assessing association of IGF-1 with follow-up cognitive measures, the corresponding cognitive measure at baseline was included as a covariate.

Additional analyses were conducted as described below. First, regression analyses of cognitive scores at follow-up with quintiles of IGF-1 were performed using the complete case dataset, as the assumption of MAR underlying the imputed dataset cannot be conclusively established. Second, the above-mentioned analysis was repeated after splitting the sample into middle-aged (< 60 years) and older (> 60 years) groups to determine whether association between IGF-1 and cognition differed in the two age groups. Third, the analyses was performed using continuous values of IGF-1 to assess for linear trends between serum IGF-1 and cognition. Finally, the analysis was performed using quartiles of serum IGF-1 values in place of quintiles to determine if the association with cognitive function at follow-up were influenced by group definitions according to quintiles.

**Fig 1:**
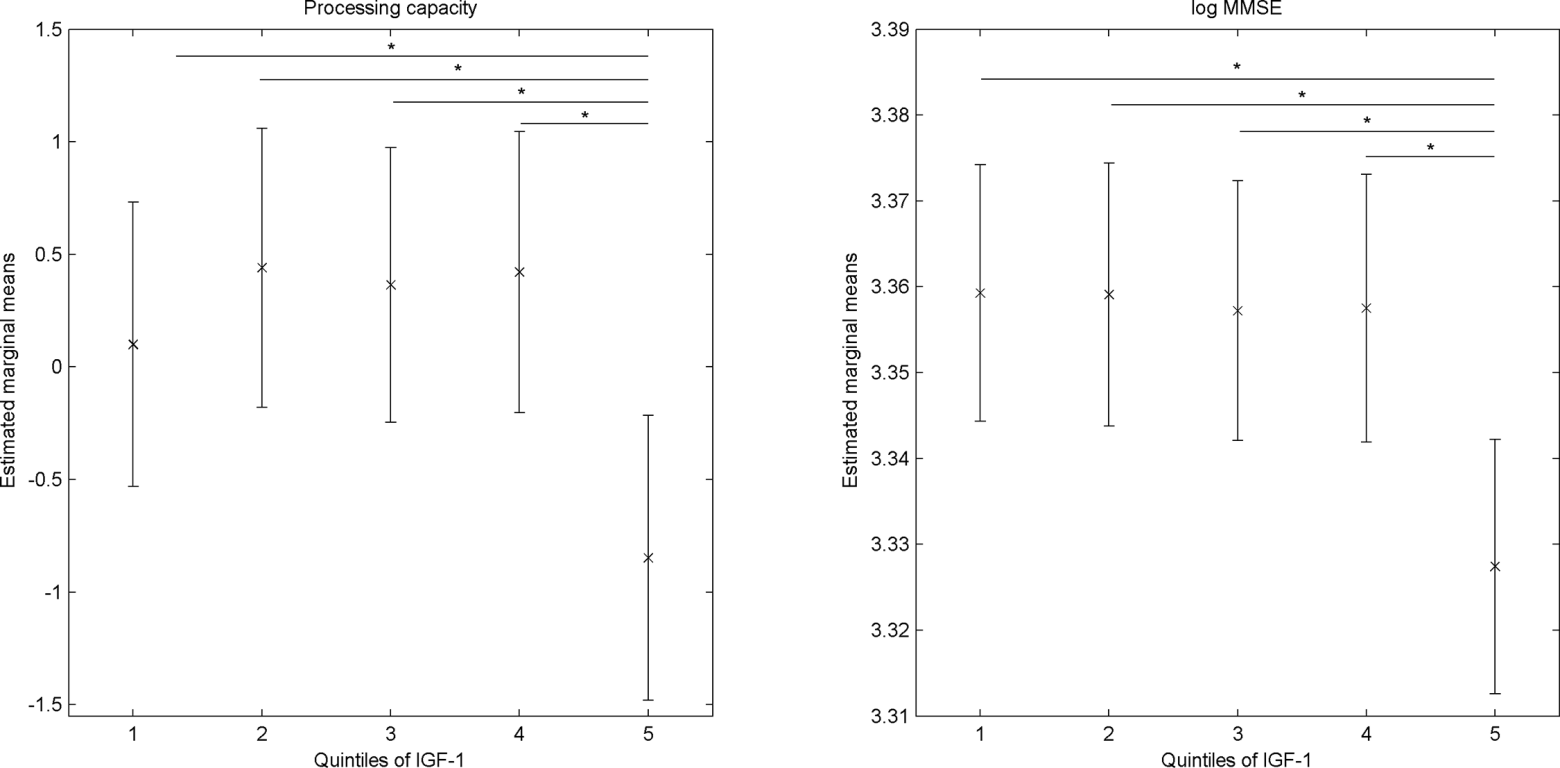
Mean performance in cognitive domains at follow-up for quintiles of IGF-1 in complete case dataset (adjusted for cognitive scores at baseline, age, education level, BMI, smoking, physical activity, and glucose levels. Error bars indicate 95% confidence intervals of the adjusted mean cognitive score. Higher values on y-axis represent better cognitive performance. Sample size in each quintile: Q1 = 53, Q2 = 53, Q3 = 56, Q4 = 52, Q5 = 51. * indicates significant at a *p* value of < .05).

Statistical analyses were conducted in R software, version 3.1.1 using the packages *car*, *effects* and *multcomp*. MI was performed using the *mice* and *miceadds* packages in R. Fig 1 was plotted using MATLAB (2011b, The MathWorks, Inc.). Results were considered significant at a two-sided value of *p* < .05.

## Results

A stratified sample of 400 middle-aged and older males composed of 100 participants in each ten-year age group from 40 to 80 years were randomly drawn from volunteers who responded to invitations to participate in this study. Letters were sent to individuals suggested by female participants of past studies at the research center and to randomly selected individuals drawn from community registers. The mean age (range) at baseline assessment was 60.2 (40–80) years. After 8 years (standard deviation: 0.4 years), neuropsychological assessment was repeated for 286 participants. The mean age (range) at follow-up was 67.2 (48–88). Table 1 presents baseline sample characteristics according to quintiles of IGF-1 of those assessed at follow-up, and Table 2 presents the mean cognitive scores of participants in each quintile at both time points. Participants assessed at follow-up were significantly younger, better educated, had lower glucose levels, and had higher scores on all cognitive domain scores and MMSE scores at baseline than those not re-assessed. Serum IGF-1 levels did not differ between those assessed and those not assessed at follow-up. Baseline characteristics of the complete sample are provided in S1 Table.

**Table 1:** Baseline characteristics and cognitive performance of participants assessed at follow-up (n = 286) according to quintiles of IGF-1.

	Quintiles of serum IGF-1 [range in ng/ml]					
	Q1	Q2	Q3	Q4	Q5	*p*
	[47–96]	[97–117]	[118–137]	[138–162]	[164–512]	
**Participants**	61	56	57	53	59	-
**IGF-1 (ng/ml)**	85.0 (10.7)	107.0 (5.7)	126.8 (5.6)	149.0 (7.0)	204.7 (56.2)	-
**Age (years)**	62.2 (10.0)	55.8 (10.3)	58.0 (11.7)	59.0 (10.0)	56.8 (11.4)	.03
**Glucose (mmol/L)**	6.1 (1.5)	5.9 (1.7)	5.6 (0.9)	5.8 (1.2)	5.7 (0.9)	.44
**BMI**	27.0 (4.3)	26.1 (2.9)	25.2 (3.1)	25.8 (3.0)	26.5 (3.4)	.02
**Smoking (pack years)**	19.1 (23.5)	13.6 (17.0)	14.9 (20.6)	16.7 (22.1)	15.6 (19.6)	.69
**Physical activity**	17.8 (8.2)	17.3 (6.6)	20.5 (7.3)	17.6 (7.5)	17.4 (6.1)	.18
**Education Level^**	4.6 (1.9)	4.9 (1.8)	5.3 (1.6)	4.9 (1.8)	4.8 (2.1)	.28

^ Verhage scale

Values given are Mean (SD) at baseline unless stated otherwise

[] represent range.

**Table 2:** Cognitive scores at baseline and follow-up.

		Q1	Q2	Q3	Q4	Q5	*p*
	Baseline						
**Memory performance**	Mean (SD)	-0.08 (2.2)	0.27 (2.0)	0.50 (2.5)	-0.81 (2.5)	-0.06 (2.8)	.85
	Range	[-7.1 to 4.5]	[-5.0 to 4.9]	[-8.5 to 5.1]	[-10.4 to 6.2]	[-7.6 to 5.1]	
**Processing capacity**	Mean (SD)	-0.14 (3.1)	0.30 (3.0)	0.39 (3.3)	-0.03 (3.1)	-0.36 (3.8)	.52
	Range	[-10.3 to 8.4]	[-7.2 to 8.7]	[-8.9 to 7.9]	[-9.4 to 7.3]	[-11.4 to 9.0]	
**Executive function**	Mean (SD)	-0.23 (3.2)	-0.24 (2.4)	0.70 (3.4)	0.04 (2.9)	-0.24 (3.4)	.25
	Range	[-8.3 to 7.2]	[-5.8 to 4.8]	[-10.4 to 8.8]	[-7.0 to 6.1]	[-10.1 to 7.0]	
**MMSE**	Mean (SD)	27.9 (1.4)	27.9 (1.3)	27.9 (1.6)	28.0 (1.6)	28.0 (1.8)	.96
	Range	[24 to 30]	[24 to 30]	[21 to 30]	[22 to 30]	[21 to 30]	
	Follow-up						
**Memory performance**	Mean (SD)	-0.61 (3.2)	0.81 (2.7)	0.47 (3.1)	0.25 (3.6)	0.25 (3.3)	.20
	Range	[-9.2 to 4.2]	[-4.8 to 6.0]	[-9.3 to 6.6]	[-11.0 to 6.1]	[-8.7 to 7.3]	
**Processing capacity**	Mean (SD)	-0.27 (3.9)	0.56 (3.1)	0.85 (3.0)	0.13 (3.5)	-0.60 (4.1)	.19
	Range	[-12.8 to 8.1]	[-7.3 to 7.1]	[-9.2 to 6.7]	[-10.4 to 7.1]	[-18.5 to 5.8]	
**Executive function**	Mean (SD)	-0.59 (3.5)	-0.22 (3.0)	1.22 (2.8)	0.22 (3.6)	0.18 (4.0)	.07
	Range	[-9.4 to 7.2]	[-7.3 to 7.0]	[-5.1 to 8.0]	[-8.2 to 7.2]	[-7.1 to 13.5]	
**MMSE**	Mean (SD)	28.6 (1.7)	28.9 (1.4)	28.9 (1.7)	28.8 (1.4)	28.0 (2.1)	.03
	Range	[21 to 30]	[25 to 30]	[20 to 30]	[24 to 30]	[23 to 30]	

Linear regressions were carried out using the imputed dataset. Results showed that quintiles of serum IGF-1 were not associated with cognitive functions at baseline (S2 Table). Linear regressions of cognitive functions at follow-up on quintiles of IGF-1 showed statistically significant negative associations with processing capacity and MMSE scores (Table 3). Results from adjusted models (i.e. controlled for age, education level, smoking, BMI, physical activity, glucose levels, and baseline cognitive score) revealed that Q2 (B = 1.04, SE = 0.47, *p* = .026) and Q3 (B = 1.02, SE = 0.5, *p* = .045) were associated with higher processing speed at follow-up than Q5. Similarly, log MMSE scores at follow-up were higher in Q1 (B = 0.03, SE = 0.01, *p* = .008), Q2 (B = 0.03, SE = 0.01, *p* = .005), Q3 (B = 0.03, SE = 0.01, *p* = .018), and Q4 (B = 0.03, SE = 0.01, *p* = .038), as compared to Q5 (Table 3). Analyses of the complete case dataset showed that results were comparable to those obtained from the imputed dataset (see S3 Table for further details). In addition, robust regression was performed on the complete case dataset to determine if outliers influence the results. The results were comparable to those from ordinary least squares regression with additional significant differences being present in memory performance (Q2 higher than Q5) and executive function (Q4 higher than Q5) (S3 Table). To visualize these results, we plotted adjusted mean scores for processing capacity and log MMSE at follow-up for each quintile (Fig 1). S1 Fig shows cognitive scores of each subject at baseline and follow-up, with and without adjustment for covariates.

**Table 3:** B (95% CI) for association between cognitive scores at follow-up and quintiles of IGF-1.

	Q1	Q2	Q3	Q4	Q5
adjusted for baseline cognition					
**Memory performance**	-0.27 (-1.20–0.66)	0.58 (-0.28 to 1.44)	0.28 (-0.65 to 1.20)	0.43 (-0.48 to 1.33)	Ref.
**Processing capacity**	0.52 (-0.54 to 1.57)	0.99^*^ (0.04 to 1.95)	0.90 (-0.12 to 1.91)	0.85 (-0.15 to 1.85)	Ref.
**Executive function**	-0.37 (-1.39 to 0.60)	-0.02 (-0.96 to 0.91)	0.15 (-0.77 to 1.07)	0.23 (-0.70 to 1.15)	Ref.
**Log MMSE scores**	0.02^*^ (<0.01 to 0.05)	0.03^**^ (0.01 to 0.06)	0.03^*^ (0.01 to 0.05)	0.03^*^ (<0.01 to 0.05)	Ref.
Fully adjusted model					
**Memory performance**	0.05 (-0.89 to 0.99)	0.61 (-0.24 to 1.45)	0.31 (-0.61 to 1.22)	0.43 (-0.46 to 1.31)	Ref.
**Processing capacity**	0.91 (-0.13 to 1.95)	1.04^*^ (0.12 to 1.96)	1.02^*^ (0.02 to 2.01)	0.92 (-0.05 to 1.88)	Ref.
**Executive function**	-0.11 (-1.05 to 0.82)	-0.11 (-0.98 to 0.75)	0.06 (-0.8 to 0.92)	0.20 (-0.65 to 1.05)	Ref.
**Log MMSE scores**	0.03^**^ (0.01 to 0.05)	0.03^**^ (0.01 to 0.05)	0.03^*^ (0.01 to 0.05)	0.03^*^ (<0.01 to 0.05)	Ref.

* significant at *p* < .05

** significant at *p* < .01

Ref. indicates reference quintile; Fully adjusted models include baseline cognitive score, age, level of education, BMI, smoking (in pack-years), physical activity, and blood glucose levels

Further analyses were carried out to determine if associations of serum IGF-1 with follow-up processing speed and MMSE scores were driven primarily by those in the older age group. Linear regressions of cognitive scores on IGF-1 quintiles were repeated after stratifying the sample based on age. Categories were defined as middle-aged (<60 years at baseline), and old (>60 years at baseline). Results showed that the association of lower cognition with high IGF-1 were not driven by the older age group. Processing capacity was no longer significantly associated with quintiles of IGF-1 in either age group. As compared to Q5, log MMSE scores were higher in Q3 (B = 0.02, SE = 0.01, *p* = .049) of the middle-aged group, and in Q2 (B = 0.05, SE = 0.02, *p* = .025) of the older age group. In addition, baseline score for processing capacity in the older age group was higher in Q3 (B = 1.22, SE = 0.6, *p* = .044), compared to Q5 (S4 Table).

Analyses of associations between continuous serum IGF-1 scores at baseline and follow-up cognition were carried out using the imputed dataset. Results showed that serum IGF-1 levels at baseline were negatively associated with follow-up MMSE scores after controlling for baseline MMSE scores and confounding factors. No association between IGF-1 on processing capacity were found (S5 Table). Lastly, we regrouped serum IGF-1 into quartiles in the imputed dataset to assess if larger values of serum IGF-1 in Q5 were driving the associations with cognitive functions. Results obtained were comparable to those with quintiles of IGF-1 (S6 Table).

## Discussion

In a longitudinal population cohort of middle-aged and older men, high levels of serum IGF-1 at baseline were associated with a decline in cognitive function after eight years. The top quintile (Q5) of IGF-1 was associated with a larger decline than in quintile 2 and 3 in processing capacity, a cognitive domain sensitive to age-related changes. Q5 was also associated with a larger decline than other quintiles in MMSE scores, a measure of global cognition. Analyses using quartiles of IGF-1 showed comparable results; the top quartile was associated with a worsening of processing capacity and MMSE scores. When analyzed as a continuous measure, serum IGF-1 was associated with a decline in MMSE scores at follow-up. No associations were found between serum IGF-1 and cognition at baseline. However, older adults (>60 years) in Q5 showed lower processing capacity at baseline as compared to quintile 3. Together, these results suggest that high serum IGF-1 levels are associated with worse future cognitive function in middle-aged and older men.

A number of epidemiological studies have reported that IGF-1 levels are positively associated with cognition [23] in domains of processing capacity [5,8], verbal memory [10], working memory [24], executive function, [25] and global cognition [8,9] in older individuals. However, a recent study did not find any evidence for associations between IGF-1 and concurrent cognition in middle-aged individuals [26], which was supported by results from the current study. In contrast to previous studies, our results indicated that high levels of IGF-1 in middle- aged and older men are associated with worse future cognitive performance and with lower concurrent processing capacity specifically in older individuals. While the contrasting results compared to previous studies may have stemmed from differences in study design and follow-up duration, the inclusion of middle-aged individuals who typically have higher IGF-1 levels than in later life [27], may have also led to a higher range of IGF-1 in the current sample. Moreover, if higher IGF-1 levels indeed indicate future cognitive decline, then previous studies in healthy older subjects may have inadvertently excluded those with high IGF-1 levels.

In some studies [8,25,28] but not all [9,24], low IGF-1 levels below 75 ng/ml were associated with poor cognition, a level present in few (n = 8) subjects in the current sample (Q1: 47–96 ng/ml). The relatively high values of IGF-1 in the lowest quintile may have contributed to the lack of associations between low IGF-1 and cognition. Further, serum IGF-1 levels greater than 106 ng/ml [25] to 118 ng/ml [28] were associated with better cognition in older adults. These values fall in the second and third quintiles in the current sample, whereas IGF-1 levels in Q5 were higher than reference levels expected for the age range of the current sample (91.1–135.7 ng/ml [29]). Together, these results indicate that the absolute level of IGF-1 may be relevant to cognition.

The characteristics of participants also differed according to quintiles of IGF-1. Those in the third quintile (118–137 ng/ml) had lower BMI and blood glucose levels, and tended to be better educated, have higher scores and lower smoking rates (S1 Table). In contrast, those in Q1 have worse scores on the same indicators, which suggests that IGF-1 levels may indicate health status in general rather than cognition only. Since the analyses were adjusted for these confounding factors, the association of IGF-1 with cognition may be independent of general health status.

The complex relationship of IGF-1 with brain function is also reflected in the heterogeneous results from studies in Alzheimer’s disease (AD), a neurodegenerative disorder characterized by declining cognition. In AD and in those at high risk to develop AD, higher IGF-1 levels have been reported [30,31,32,33]. However, lower IGF-1 levels in association with AD have also been reported [34,35,36]. Moreover, raising serum IGF-1 levels was reported to improve cognition in those at high risk for AD [37]. The contrasting results highlight the difficulty in conclusively establishing the effects of IGF-1 on cognition despite its fundamentally essential role in the brain [38]. That poor cognition is associated with lower (from past studies) and higher (from current study) levels of IGF-1 suggests that cognitive function may benefit from optimum levels of IGF-1, similar to associations with hormonal levels such as testosterone [17,39].

While the mechanism for the association between high IGF-1 and cognition are not clear, studies suggest a role for the insulin/IGF signaling (IIS) system [40]. Lower activity in the IIS resulting from polymorphisms in IGF-1 receptor genes has been associated with longevity in centenarians [41] as well as with lower risk for cognitive decline in those older than 85 years [42], suggesting that low IIS activity may be especially beneficial for longevity and cognition. We speculate that increased IGF-1 levels lead to a sustained increase in IIS, which in turn leads to deleterious effects that speed up cellular aging [43]. An alternative explanation may be that raised serum IGF-1 levels reflect a compensatory response to ongoing deleterious changes. These speculations need confirmation in future studies.

While interpreting the results the following limitations must be considered. First, serum IGF-1 levels were not assessed at follow-up. It may be possible that poor cognition at follow-up is associated with change in IGF-1 between the two visits rather than with baseline levels. Second, free forms of IGF-1 and IGF binding proteins, which may be associated with cognitive function, were not assessed in this study. Third, these results may not be generalizable to females considering that associations of serum IGF-1 with behavioral symptoms show gender differences [44]. Lastly, serum levels of IGF-1 are known to differ from that available locally in the brain [45] and hence serum IGF-1 may not reliably indicate IGF-1 activity in the brain.

To conclude, serum IGF-1 is not associated with concurrent cognitive function in middle-aged males, while older males with high IGF-1 show poor concurrent cognition. In both middle-aged and older males, high levels of IGF-1 beyond a threshold are associated with a decline in future cognitive function. We suggest that optimum levels of IGF-1 may be associated with better long term cognitive function. Further studies are needed to determine the absolute levels of IGF-1, which may be associated with poor cognition.

## Supporting Information

S1 Data Imputed dataset generated with 30 imputations. (DAT)

S1 Fig Cognitive performance at baseline and follow-up. Upper panel represents cognitive performance of each subject (on Y-axis) at baseline (T1) and follow-up (T2) connected by a line. Lower panel represents adjusted cognitive performance for each subject, controlling for age, education level, BMI, smoking, physical activity, and glucose levels at both visits. Thick black line indicates the mean change in cognitive performance between visits and the grey band represents 95% confidence intervals.
(TIF)

S1 Table Baseline characteristics and cognitive performance of complete sample (n = 400) according to quintiles of IGF-1. (DOCX)

S2 Table B (95% CI) for association between baseline cognitive scores and quintiles of IGF-1. (DOCX)

S3 Table Associations between follow-up cognitive scores and quintiles of IGF-1 in complete case dataset. (DOCX)

S4 Table B (95% CI) for association between cognitive scores at follow-up, and at baseline with quintiles of IGF-1, separated by age classes. (DOCX)

S5 Table B (95% CI) for association between cognitive scores and serum levels of IGF-1. (DOCX)

S6 Table B (95% CI) for association between follow-up cognitive scores and quartiles of IGF-1 in the imputed dataset. (DOCX)
