# Supplementary figures and images for: Association between Cognition and Serum Insulin-Like Growth Factor-1 in Middle-Aged & Older Men: An 8 Year Follow-Up Study
Source: PLoS One. 2016 Apr 26;11(4):e0154450. doi: 10.1371/journal.pone.0154450 (PMC4846160; doi:10.1371/journal.pone.0154450)

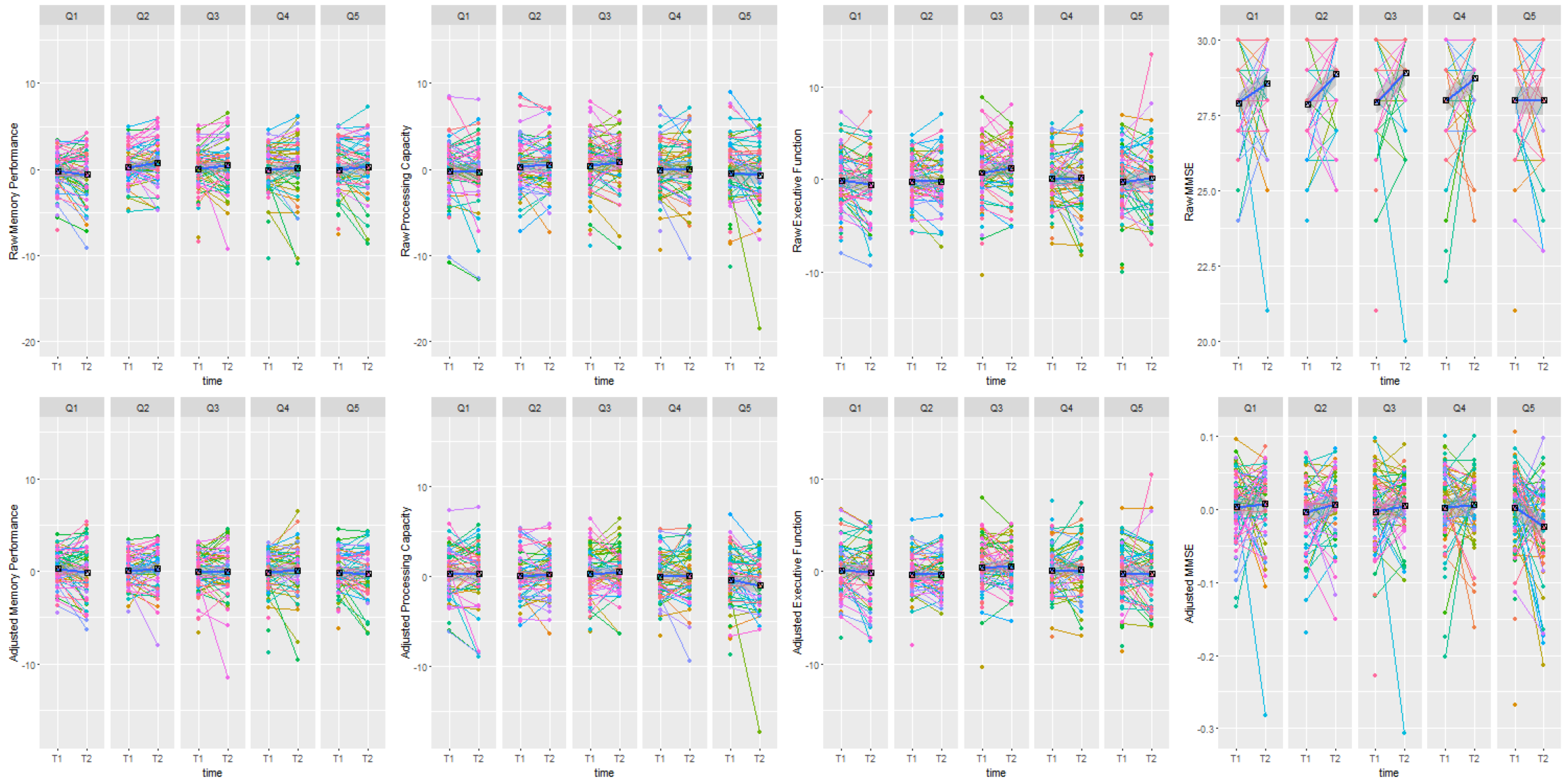

Supplement: S1 Fig — Upper panel represents cognitive performance of each subject (on Y-axis) at baseline (T1) and follow-up (T2) connected by a line. Lower panel represents adjusted cognitive performance for each subject, controlling for age, education level, BMI, smoking, physical activity, and glucose levels at both visits. Thick black line indicates the mean change in cognitive performance between visits and the grey band represents 95% confidence intervals. (TIF) [file pone.0154450.s002.tif]
