# Supplementary material for: Association between Cognition and Serum Insulin-Like Growth Factor-1 in Middle-Aged & Older Men: An 8 Year Follow-Up Study
Source: PLoS One. 2016 Apr 26;11(4):e0154450. doi: 10.1371/journal.pone.0154450 (PMC4846160; doi:10.1371/journal.pone.0154450)
Supplement: S1 Table — (DOCX) [file pone.0154450.s003.docx]

**S1 Table:** Baseline characteristics and cognitive performance of participants assessed at baseline (n=400) according to quintiles of IGF-1

|  | **Quintiles of serum IGF-1 [range in ng/ml]** | | | | |  |
| --- | --- | --- | --- | --- | --- | --- |
|  | **1** | **2** | **3** | **4** | **5** | ***p*** |
|  | **[44-96]** | **[97-117]** | **[118-137]** | **[138-162]** | **[163-512]** |  |
| **Participants** | 85 | 75 | 80 | 80 | 80 | - |
| **IGF-1 (ng/ml)** | 84.1 (11.5) | 106.7 (5.6) | 127.1 (5.8) | 149.3 (7.0) | 201.7 (49.3) | - |
| **Age (years)** | 63.7 (10.1) | 58.1 (10.6) | 60.1 (12.5) | 59.6 (10.7) | 59.3 (11.9) | .02 |
| **Glucose (nmol/L)** | 6.4 (2.1) | 6.1 (1.7) | 5.7 (0.9) | 5.8 (1.1) | 5.8 (0.9) | .01 |
| **BMI** | 27.0 (4.4) | 26.5 (3.0) | 25.3 (3.1) | 26.1 (3.3) | 26.4 (3.2) | .02 |
| **Smoking (pack years)** | 21.4 (25.0) | 14.6 (16.6) | 14.5 (20.1) | 16.8 (21.1) | 15.9 (19.0) | .19 |
| **Physical activity** | 17.0 (8.2) | 17.2 (7.5) | 19.6 (7.2) | 18.3 (6.3) | 17.1 (8.2) | .23 |
| **Education Level^** | 4.7 (2.0) | 4.7 (1.9) | 5.0 (1.7) | 4.6 (1.8) | 4.6 (2.1) | .68 |

IGF-1: Insulin like Growth Factor-1; BMI: Body Mass Index; ^ Verhage scale; Values given are Mean (SD) at baseline unless stated otherwise.
