# Supplementary material for: Association between Cognition and Serum Insulin-Like Growth Factor-1 in Middle-Aged & Older Men: An 8 Year Follow-Up Study
Source: PLoS One. 2016 Apr 26;11(4):e0154450. doi: 10.1371/journal.pone.0154450 (PMC4846160; doi:10.1371/journal.pone.0154450)
Supplement: S2 Table — (DOCX) [file pone.0154450.s004.docx]

**S2 Table:** B (95% CI) for association between baseline cognitive scores and quintiles of IGF-1

|  | **Q1** | **Q2** | **Q3** | **Q4** | **Q5** |
| --- | --- | --- | --- | --- | --- |
| Unadjusted models | | | | | |
| **Memory performance** | -0.07  (-0.81 to 0.67) | 0.32  (-0.44 to 1.08) | 0.11  (-0.64 to 0.86) | -0.02  (-0.77 to 0.73) | Reference |
| **Processing capacity** | 0.13  (-0.88 to 1.15) | 0.66  (-0.38 to 1.71) | 0.75  (-0.27 to 1.78) | 0.34  (-0.69 to 1.36) | Reference |
| **Executive function** | -0.05  (-1.01 to 0.91) | -0.02  (-1.01 to 0.96) | 0.94  (-0.03 to 1.90) | 0.28  (-0.69 to 1.25) | Reference |
| **Log MMSE scores** | -0.004  (-0.02 to 0.01) | -0.004  (-0.02 to 0.01) | -0.003  (-0.02 to 0.02) | -0.001  (-0.02 to 0.02) | Reference |
| Adjusted model | | | | | |
| **Memory performance** | 0.41  (-0.20 to 1.02) | 0.19  (-0.43 to 0.81) | 0.06  (-0.55 to 0.67) | 0.04  (-0.57 to 0.64) | Reference |
| **Processing capacity** | 0.60  (-0.23 to 1.43) | 0.49  (-0.35 to 1.33) | 0.65  (-0.18 to 1.48) | 0.41  (-0.41 to 1.23) | Reference |
| **Executive function** | 0.20  (-0.65 to 1.05) | -0.09  (-0.95 to 0.76) | 0.65  (-0.20 to 1.50) | 0.32  (-0.53 to 1.15) | Reference |
| **Log MMSE scores** | -0.0004  (-0.02 to 0.02) | -0.0060  (-0.02 to 0.01) | -0.0060  (-0.02 to 0.01) | -0.0010  (-0.02 to 0.02) | Reference |

Adjusted models include age, level of education, BMI, smoking, physical activity, and glucose levels; MMSE: mini mental state examination; BMI: body mass index
