# Supplementary material for: Association between Cognition and Serum Insulin-Like Growth Factor-1 in Middle-Aged & Older Men: An 8 Year Follow-Up Study
Source: PLoS One. 2016 Apr 26;11(4):e0154450. doi: 10.1371/journal.pone.0154450 (PMC4846160; doi:10.1371/journal.pone.0154450)
Supplement: S3 Table — (DOCX) [file pone.0154450.s005.docx]

**S3 Table:**

(i) B (95% CI) for association between follow-up cognitive scores and quintiles of IGF-1 in complete case dataset

|  | **Q1** | **Q2** | **Q3** | **Q4** | **Q5** |
| --- | --- | --- | --- | --- | --- |
| **Memory performance** | 0.11  (-0.74 to 0.96) | 0.80  (-0.03 to 1.64) | 0.41  (-0.44 to 1.26) | 0.45  (-0.41 to 1.31) | Reference |
| **Processing capacity** | 0.95^*^  (0.05 to 1.85) | 1.29^**^  (0.41 to 2.17) | 1.21^**^  (0.33 to 2.10) | 1.27^**^  (0.38 to 2.16) | Reference |
| **Executive function** | 0.07  (-0.88 to 1.02) | 0.16  (-0.77 to 1.08) | 0.22  (-0.72 to 1.15) | 0.55  (-0.38 to 1.49) | Reference |
| **Log MMSE scores** | 0.03^**^  (0.01 to 0.05) | 0.03^**^  (0.01 to 0.05) | 0.03^**^  (0.01 to 0.05) | 0.03^**^  (0.01 to 0.05) | Reference |

* significant at *p* < .05, ** significant at *p* < .01; Models adjusted for baseline cognitive score, age, level of education, BMI, smoking, physical activity, and glucose levels. MMSE: mini mental state examination; BMI: body mass index

(ii) B (95% CI) as determined by robust regression for association between follow-up cognitive scores and quintiles of IGF-1 in complete case dataset

|  | **Q1** | **Q2** | **Q3** | **Q4** | **Q5** |
| --- | --- | --- | --- | --- | --- |
| **Memory performance** | 0.17  (-0.70 to 1.04) | 0.91^*^  (0.16 to 1.66) | 0.59  (-0.19 to 1.36) | 0.63  (-0.18 to 1.36) | Reference |
| **Processing capacity** | 0.91^*^  (0.06 to 1.76) | 1.02^**^  (0.30 to 1.75) | 0.83^*^  (0.02 to 1.64) | 0.95^*^  (0.11 to 1.78) | Reference |
| **Executive function** | 0.51  (-0.39 to 1.41) | 0.55  (-0.29 to 1.38) | 0.55  (-0.30 to 1.40) | 0.94^*^  (0.04 to 1.83) | Reference |
| **Log MMSE scores** | 0.02^*^  (0.01 to 0.04) | 0.02^**^  (<0.01 to 0.04) | 0.02^**^  (0.01 to 0.04) | 0.02^*^  (<0.01 to 0.04) | Reference |

* significant at *p* < .05, ** significant at *p* < .01; Models adjusted for baseline cognitive score, age, level of education, BMI, smoking, physical activity, and glucose levels. MMSE: mini mental state examination; BMI: body mass index
