# Supplementary material for: Association between Cognition and Serum Insulin-Like Growth Factor-1 in Middle-Aged & Older Men: An 8 Year Follow-Up Study
Source: PLoS One. 2016 Apr 26;11(4):e0154450. doi: 10.1371/journal.pone.0154450 (PMC4846160; doi:10.1371/journal.pone.0154450)
Supplement: S4 Table — (DOCX) [file pone.0154450.s006.docx]

**S4 Table:** B (95% CI) for association between cognitive scores at follow-up, and at baseline with quintiles of IGF-1, separated by age classes

|  |  | **Q1** | | **Q2** | **Q3** | **Q4** | | **Q5** |
| --- | --- | --- | --- | --- | --- | --- | --- | --- |
|  | **Adjusted models for cognitive scores at follow-up** | | | | | | | |
| **Memory performance** | < 60yrs | -0.02  (-1.10 to 1.07) | | 0.45  (-0.52 to 1.42) | 0.42  (-0.60 to 1.44) | | 0.52  (-0.56 to 1.60) | Ref. |
|  | > 60yrs | -0.25  (-1.72 to 1.22) | | 0.50  (-1.13 to 2.13) | -0.09  (-1.46 to 1.27) | | -0.04  (-1.50 to 1.43) | Ref. |
| **Processing capacity** | < 60yrs | 0.22  (-0.82 to 1.26) | | 0.44  (-0.46 to 1.35) | 0.51  (-0.42 to 1.43) | | 0.30  (-0.63 to 1.23) | Ref. |
|  | > 60yrs | 1.46  (-0.45 to 3.36) | | 1.78  (-0.28 to 3.84) | 1.60  (-0.30 to 3.50) | | 1.52  (-0.35 to 3.39) | Ref. |
| **Executive function** | < 60yrs | -0.15  (-1.34 to 1.04) | | -0.06  (-1.10 to 0.97) | -0.21  (-1.31 to 0.88) | | 0.56  (-0.58 to 1.70) | Ref. |
|  | > 60yrs | 0.49  (-0.95 to 1.92) | | 0.38  (-1.27 to 2.02) | 0.93  (-0.55 to 2.41) | | 0.21  (-1.13 to 1.53) | Ref. |
| **Log MMSE scores** | < 60yrs | 0.020  (-0.0030 to 0.0500) | | 0.020  (-0.0040 to 0.0400) | 0.020^*^  (0.0003 to 0.0400) | | 0.010  (-0.0100 to 0.0400) | Ref. |
|  | > 60yrs | 0.040  (-0.0030 to 0.0800) | | 0.050^*^  (0.0060 to 0.0900) | 0.030  (-0.0100 to 0.0800) | | 0.040  (-0.0100 to 0.0800) | Ref. |
|  | **Adjusted models for cognitive scores at baseline** | | | | | | | |
| **Memory performance** | < 60yrs | | -0.22  (-1.08 to 0.64) | -0.33  (-1.07 to 0.41) | -0.60  (-1.37 to 0.16) | | -0.25  (-1.01 to 0.52) | Ref. |
|  | > 60yrs | | 0.85  (-0.07 to 1.76) | 0.71  (-0.33 to 1.75) | 0.71  (-0.27 to 1.68) | | 0.26  (-0.71 to 1.23) | Ref. |
| **Processing capacity** | < 60yrs | | 0.52  (-0.81 to 1.84) | 0.13  (-1.01 to 1.28) | 0.10  (-1.08 to 1.28) | | 0.12  (-1.05 to 1.30) | Ref. |
|  | > 60yrs | | 0.87  (-0.25 to 1.99) | 0.86  (-0.41 to 2.14) | 1.22^*^  (0.03 to 2.41) | | 0.84  (-0.35 to 2.02) | Ref. |
| **Executive function** | < 60yrs | | -0.26  (-1.49 to 0.96) | -0.06  (-1.11 to 1.00) | 0.80  (-0.29 to 1.89) | | 0.25  (-0.83 to 1.34) | Ref. |
|  | > 60yrs | | 0.31  (-0.95 to 1.57) | -0.35  (-1.78 to 1.08) | 0.51  (-0.83 to 1.85) | | 0.30  (-1.03 to 1.63) | Ref. |
| **Log MMSE scores** | < 60yrs | | -0.010  (-0.0300 to 0.0100) | -0.020  (-0.0300 to 0.0040) | - 0.010  (-0.0300 to 0.0060) | | -0.010  (-0.0300 to 0.0100) | Ref. |
|  | > 60yrs | | 0.008  (-0.0200 to 0.0300) | 0.004  (-0.0300 to 0.0300) | 0.004  (-0.0200 to 0.0300) | | 0.007  (-0.0200 to 0.0400) | Ref. |

*significant at *p* < .05; Adjusted models include age, level of education, BMI, smoking, physical activity, and glucose levels; Follow-up models additionally adjusted for baseline cognitive score; MMSE: mini mental state examination; BMI: body mass index
