# Supplementary material for: Association between Cognition and Serum Insulin-Like Growth Factor-1 in Middle-Aged & Older Men: An 8 Year Follow-Up Study
Source: PLoS One. 2016 Apr 26;11(4):e0154450. doi: 10.1371/journal.pone.0154450 (PMC4846160; doi:10.1371/journal.pone.0154450)
Supplement: S5 Table — (DOCX) [file pone.0154450.s007.docx]

**S5 Table:** B (95% CI) for association between cognitive scores and serum levels of IGF-1

|  | **Unadjusted models** | | | | **Adjusted models** | |
| --- | --- | --- | --- | --- | --- | --- |
|  | **Baseline** | | **Follow-up^1^** | | **Baseline** | **Follow-up** |
| **Memory performance** | -0.0010  (-0.006 to 0.005) | 0.0200  (-0.004 to 0.008) | | -0.0030  (-0.007 to 0.001) | | -0.0002  (-0.006 to 0.006) |
| **Processing capacity** | -0.002  (-0.009 to 0.005) | -0.002  (-0.009 to 0.005) | | -0.004  (-0.010 to 0.001) | | -0.005  (-0.012 to 0.002) |
| **Executive function** | 0.002  (-0.004 to 0.009) | 0.003  (-0.003 to 0.010) | | 0.001  (-0.005 to 0.007) | | 0.002  (-0.004 to 0.008) |
| **Log MMSE scores** | 0.00004  (-0.00009 to 0.00010) | -0.00020^*^  (-0.00030 to -0.00001) | | 0.00002  (-0.00009 to 0.00010) | | -0.00020^**^  (-0.00040 to -0.00006) |

^1^Adjusted for baseline cognitive score; * significant at *p* < .05, ** significant at *p* < .01; Adjusted models include baseline cognitive score, age, level of education, BMI, smoking, physical activity, and glucose levels. MMSE: mini mental state examination; BMI: body mass index
