# Supplementary material for: Association between Cognition and Serum Insulin-Like Growth Factor-1 in Middle-Aged & Older Men: An 8 Year Follow-Up Study
Source: PLoS One. 2016 Apr 26;11(4):e0154450. doi: 10.1371/journal.pone.0154450 (PMC4846160; doi:10.1371/journal.pone.0154450)
Supplement: S6 Table — (DOCX) [file pone.0154450.s008.docx]

**S6 Table:** B (95% CI) for association between follow-up cognitive scores and quartiles of IGF-1 in the imputed dataset

|  | **Q1**  **[44 - 101]** | **Q2**  **[102 – 126]** | **Q3**  **[127 - 155]** | **Q4**  **[156 - 512]** |
| --- | --- | --- | --- | --- |
| **n** | 102 | 98 | 100 | 100 |
| **Memory performance** | -0.09  (-0.88 to 0.70) | 0.30  (-0.49 to 1.09) | 0.04  (-0.76 to 0.84) | Reference |
| **Processing capacity** | 0.72  (-0.18 to 1.62) | 1.07^*^  (0.23 to 1.91) | 0.83^*^  (0.03 to 1.63) | Reference |
| **Executive function** | -0.20  (-1.02 to 0.62) | -0.05  (-0.83 to 0.73) | 0.09  (-0.68 to 0.86) | Reference |
| **Log MMSE scores** | 0.03^*^  (0.010 to 0.050) | 0.03^**^  (0.010 to 0.050) | 0.02^*^  (0.003 to 0.040) | Reference |

* significant at *p* < .05, ** significant at *p* < .01; Models adjusted for baseline cognitive score, age, level of education, BMI, smoking, physical activity, and glucose levels. MMSE: mini mental state examination; BMI: body mass index
